# Sense of coherence as a mediator of health-related quality of life dimensions in patients with breast cancer: a longitudinal study with prospective design

**DOI:** 10.1186/s12955-015-0392-4

**Published:** 2015-12-09

**Authors:** Camelia Rohani, Heidar-Ali Abedi, Kay Sundberg, Ann Langius-Eklöf

**Affiliations:** Department of Nursing, School of Nursing and Midwifery, Shahid Beheshti University of Medical Sciences, Vali-Asr Avenue, Cross of Vali-Asr and Neiaiesh Highway, Opposite to Rajaee Heart Hospital, Tehran, 1996835119 Iran; Department of Nursing, Nursing and Midwifery School, Islamic Azad University, Isfahan (Khorasgan) Branch, Isfahan, 8153653791 Iran; Department of Neurobiology, Care Sciences and Society, Karolinska Institutet, Alfred Nobels Alle’ 23, Huddinge, 14183 Sweden

**Keywords:** Breast cancer, Health-related quality of life, Sense of coherence, Mediator, Well-being

## Abstract

**Background:**

In our previous study, we found that the degree of sense of coherence (SOC) and baseline ratings of several dimensions of health-related quality of life (HRQoL) were the most important predictors of HRQoL changes 6 months after the pre-diagnosis period of breast cancer. To find a way to explain these findings, the aim of this study was to explore the mediating effect of the SOC between ratings of HRQoL dimensions before final diagnosis, and ratings of the same dimensions at the 6 months follow up, within a sample of women with breast cancer.

**Methods:**

A longitudinal study with a prospective design at baseline (T1) and 6 months later (T2) was conducted on 162 women with breast cancer. To measure HRQoL dimensions three different questionnaires, the European Organization for Research and Treatment of Cancer QLQ-30, the SF-12 Health Survey version 2 and the Health Index were applied at T1 and T2 to cover both diagnostic-specific and generic dimensions. Measurement of the SOC as a mediator was done by the SOC-13 scale.

**Results:**

Mediational analyses on eight significant pairs of HRQoL dimensions showed that the degree of SOC totally mediated variations of global quality of life (*p* < 0.001) as well as cognitive and social functioning (*p* <0.05) scores between T1 to T2. Changes in the scores of emotional functioning (*p* < 0.01), fatigue (*p* < 0.05), financial difficulties (*p* < 0.05), well-being (*p* < 0.001), and mental health component (*p* < 0.001) were partially mediated. The degree of SOC explained 16 % to 45 % of the variances in HRQoL dimensions at T2.

**Conclusions:**

The mediating pathway of the SOC in the context of this study appears to be the key to understanding how a higher sense of coherence as an inner resource may serve as a protective psychological factor in the adaptation process of the patients. Clinicians might consider coherence-oriented structure of the SOC and the connection between the SOC and HRQoL data in intervention plans from the first visit onwards. It may assist the identification of women who are at greater risk for maladaptation to the breast cancer trajectory.

## Background

There is universal agreement on the individual and the multidimensional nature of health-related quality of life (HRQoL) [1]. HRQoL focuses on the patient’s perception of well-being, functioning and symptoms in relation to a disease and its treatment [1–3]. Most commonly HRQoL refers to the subjective experience of global health status, which is measured by patient reported outcome questionnaires [2]. Based on the World Health Organization definition, HRQoL includes at least dimensions of physical, psychological and social functioning in the context of disease [4]. Furthermore, it can be measured by both generic and disease-specific questionnaires [1]. To be diagnosed with breast cancer is stressful, and might be followed by short or long-term physical and psychological challenges with regard to treatment side effects and fear of recurrence [5, 6]. A variety of HRQoL studies of patients with cancer indicate that how a person rates his/her HRQoL at one-time point is directly related to the person’s rating at follow-ups, i.e., patients' baseline quality of life scores are predictive of the level of subsequent quality of life assessments [7–11]. Thus, higher numbers of perceived functioning impairments and symptoms at baseline (often at the time of diagnosis) are followed by the same pattern at follow-ups, irrespectively of the time frame (6, 12 or 24 months after baseline). There is also evidence that a person’s rating of his/her HRQoL is related to the person's ability to cope with and manage the challenges that may come with an illness [1, 12, 13].

Antonovsky [14] developed the concept of sense of coherence (SOC) to be an orientation to life which has an influence on health and, as an internal resource, can be helpful in managing life stressors to assist successful coping. Antonovsky [14] operationalized the concept of SOC to be measured in a 13-item scale, which has shown cross-cultural validity and reliability [15]. Three elements are included in the concept of SOC: comprehensibility (a belief that things happening in life are rational, predictable and understandable), manageability (a belief that people have the ability and the resources necessary to take care of things, and that things are manageable and within their control), and meaningfulness (a belief that things in life are worthwhile and that there is good reason to care about what happens) [14, 16]. The SOC is evidently more concentrated on factors promoting health, rather than factors causing particular diseases [14, 17]. Antonovsky [14] hypothesized a high SOC to be a salutogenic resource (creating positive health), which develops with increasing age and is related to generalized resistance resources (GRRs). The term GRRs refer to a number of resources which are bound to the person, his/her capacity and his/her environment. The GRRs are created by life experiences, and include both genetic and psychosocial dispositions, such as ego identity (strength), knowledge, intelligence, wealth, social support, cultural stability and religion, all of which makes energy available to cope with stressors [14, 16]. When the GRRs are insufficient, the person does not cope well and this may result in generalized resistance deficits (GRDs). The SOC is formed by both GRRs and GRDs and one’s own life experience [18]. As postulated by Antonovsky, the SOC mediates the effects of external stressors and resources on psychological dysfunctions [19]. Antonovsky [14] believed that the SOC concept is broader than being a specific coping strategy and that a higher SOC is required for successful overall coping and should be regarded as an ability to find and utilize resources. Antonovsky [14] discussed high and low degrees of SOC, but he didn’t define what can be considered as a normal SOC. Also, he described that the SOC is entirely developed by the end of the third decade of life, and thereafter is showing only minor and temporary fluctuations in response to stressful situations [14]. Later studies by other researchers put this issue under discussion and proposed that the SOC may not be as stable as Antonovsky said [20, 21], while others show it to be stable [22, 23]. The role of SOC as a significant predictor of HRQoL outcomes is supported in previous and current studies, i.e., the higher the SOC, the better the HRQoL [24–26], especially in psychosocial dimensions [27]. This has also been evident in studies among patients with breast cancer [11, 13, 28]. Furthermore, a systematic review of the SOC scale discussed the mediating or moderating effect of the SOC on the patients’ perceptions of their subjective health, showing that the stronger the SOC, the lower was the number of reported health impairments and symptoms [17].

The SOC scale score has shown a mediating effect in numerous studies within different samples [29–38]. Overall, these studies show that the degree of SOC mediates correlation between management of stress/symptoms and perception of health and psychological well-being. On the contrary, Lundberg [39] did not find that SOC mediates the effect of childhood factors on adult health in a Swedish population. But, in another study [40] the degree of SOC functioned as both a mediator and moderator between the experience of psychosocial work environment and the experience of stress symptoms. The results showed that persons with higher SOC might cope more efficiently with work environmental strain, than individuals with lower SOC.

In our previous study [11], we found that the degree of SOC and baseline ratings of several dimensions of HRQoL were the most important predictors of HRQoL changes 6 months after the pre-diagnosis period of breast cancer. These findings led us to test the longitudinal role of the SOC scale score as a mediator of HRQoL dimensions. A mediator shows evidence regarding casual relationship between the variables [41] or explains how or why the variables are mainly correlated [42]. To our knowledge there are no studies on the role of the SOC as a mediator between baseline levels of HRQoL dimensions and the levels of the same dimensions at follow-ups in patients with breast cancer. Therefore, based on prior experience and previous literature we hypothesized that baseline levels of HRQoL dimensions have a direct and indirect effect on the levels of the same dimensions 6 months later. The aim of this study was to explore the mediating effect of the SOC between ratings of HRQoL dimensions before final diagnosis, and ratings of the same dimensions at the 6-month follow up, within a sample of women with breast cancer. In this study, HRQoL was measured by a specific questionnaire for cancer patients, the European organization for research and treatment of cancer (EORTC) QLQ-C30 [43], and two generic questionnaires, the SF-12 Health Survey version 2 (SF-12v2) [44] and the Health Index (HI) [45]. SOC was measured by the SOC-13 scale [45]. We discuss the role of the SOC as a total or partial mediator between ratings of HRQoL dimensions at baseline (pre-diagnosis phase) and 6 months later.

## Methods

### Design

This is a longitudinal study with a prospective design by two measurement points: baseline (T1) (pre-diagnosis phase of breast cancer) and 6 months later (T2).

### Participants

Before surgery and final diagnosis of breast cancer, 254 eligible women suspected of breast cancer, with an operable lump or other symptoms in the breast, were recruited from the surgical wards at two hospitals in Tehran belonging to the Tehran University of Medical Sciences. The breast cancer diagnosis was confirmed with a quick pathology report during surgery. This report was thereafter controlled in detail, and the final result was given to the patients two to three weeks later. Women with a confirmed diagnosis of breast cancer were included in the study’s follow-up. Of these 254 women, 39 (15 %) were later diagnosed with benign tumors, 15 (6 %) did not return the questionnaires, and 10 (4 %) did not complete the questionnaires, leaving 190 participants (75 %) at T1 who had a confirmed breast cancer diagnosis after surgery. There was a further drop-out rate of 28 participants (11 %) during the 6-month follow up at T2, 23 (9 %) declined further participation, 4 (2 %) had a change of address and 1 (0.4 %) had deceased, leaving a final sample of 162 patients (64 %) that participated at both T1 and T2. Inclusion criteria were to have sufficient knowledge of the Persian language to answer the questionnaires and no previous cancer history.

### Instruments

To measure HRQoL dimensions three different questionnaires, including the EORTC QLQ-C30, the SF-12v2 and the HI were applied at T1 and T2 to cover both diagnostic-specific and generic dimensions, along with the SOC scale.

The European Organization for Research and Treatment of Cancer QLQ-30 (EORTC QLQ-C30) version 3 is a cancer-specific questionnaire that has been translated and validated in the Persian language [43]. The EORTC QLQ-C30 (30 items) comprises a global health status/quality of life scale, five functional scales (physical, PF; role, RF; emotional, EF; cognitive, CF; and social, SF), three symptom scales (fatigue, FA; pain, PA; and nausea/vomiting, NV), and six single-item scales (appetite loss, AP; insomnia, SL; dyspnea, DY; constipation, CO; diarrhea, DI; and financial difficulties, FI). The EORTC QLQ-C30 is rated on a four-point scale from 1 to 4 (except for global health status/ quality of life in which a seven-point scale is used). All of the scales are linearly transformed to a scale from 0 to 100. A high score for the global health status/ quality of life and functional scales represents a high quality of life and a healthy level of functioning. However, a high score for symptom scales or single items represents a high level of problems. The psychometric properties of this questionnaire have been supported in different countries [46], also in Iran [43]. In the present study Cronbach’s alpha coefficients for the scales of the questionnaire at T1 were >0.60, except for three scales: RF (0.46), CF (0.44), and NV (0.44). However, Cronbach’s alpha coefficients for all scales of the EORTC QLQ-C30 were greater than 0.60 at the 6-month follow-up.

The SF-12 Health Survey version 2 (SF-12v2) is a generic questionnaire, measuring subjective health and the translated Persian version of the SF-12v2 was used in this study [44]. The SF-12v2 consists of 12 items aggregated in two summary components: a Physical Component Summary (PCS) and a Mental Component Summary (MCS) which are standardized to produce a mean of 50 with a standard deviation of 10 (norm-based scoring) [47]. The higher the score, the better the perceived health of the participants [47]. In the present study Cronbach’s alpha coefficients for two summary components of the Persian version of the SF-12v2 at T1 and T2 were 0.72 and 0.78, respectively.

The Health Index (HI) is a generic questionnaire, reflecting general well-being and the validated Persian version was used [45]. It contains of nine items (energy, temper, fatigue, loneliness, sleep, dizziness, bowel function, pain and mobility) [48]. The items are rated on a verbal scale from very poor (1) to very good (4), providing a total score from 9 to 36. The higher the score, the better the individual’s perceived well-being [48]. In this study Cronbach’s alpha coefficient at T1 was 0.62 and 0.82 at T2.

The Sense of Coherence (SOC) Scale measures an individual’s global view of life based on how comprehensible, manageable, and meaningful life appears in 13 items. The respondents indicate agreement or disagreement on a seven-point semantic scale, with two anchoring responses. The total score is from 13 to 91. The higher the score, the stronger the SOC [14]. The scale is applicable cross-culturally and has acceptable validity and reliability [15]. The Persian validated version of the SOC was used in this study [45]. Cronbach’s alpha coefficient was 0.83.

### Demographic and clinical data

Demographic information and clinical data were obtained by a short interview and medical records, respectively at T1.

### Data collection procedures

Baseline data were collected at the surgical wards, on days 1–14 before a final diagnosis of breast cancer. The questionnaires comprised the EORTC QLQ-C30, the SF-12v2, the HI and the SOC scale. The 6-month follow-up data was collected by sending the same questionnaires with a letter of explanation and a pre-stamped envelope to the participants.

This study was approved by the Ministry of Health and Medical Education of Iran (The National Ethical Board of Research: P/391-31). All participants received verbal and written information, and written informed consent was obtained. All participants were in a vulnerable condition before final diagnosis of breast cancer. Thus, the researcher emphasized voluntariness and the right to withdraw from further participation at any time of the study. (License agreements #25762, #36170 permission for application of the SF-12v2 was obtained from QualityMetric Incorporated).

### Statistical analyses

All statistical analyses were conducted using the SPSS version 20. The accepted level of internal consistency of the scales was a Cronbach’s alpha above 0.60 [1]. The level of statistical significance was set at *p* <0.05. The baseline values of all scale scores of the EORTC QLQ-C30, the two summary components of the SF-12v2 (PCS and MCS) and the HI were defined as independent variables, and the same scale scores at the 6-month follow up as dependent. All variables met the normality assumptions by P-P plots. Analyses started with the calculation of Pearson product–moment correlation coefficients between the mediator (SOC scale scores), the independent (T1 scale scores), and the dependent (T2 scale scores) variables in a correlation matrix. Based on the assumption of mediation analysis [42] significant pairs (between the mediator and the independent variable as well as between the independent and the dependent variable) of the correlated variables were extracted to be entered in the Multiple Linear Regression (MLR) analyses.

**Fig. 1:**
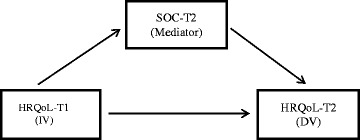
A general mediation model. The diagram does not include the control variables of age, educational level and cancer stage, but they were included in the multiple linear regression analyses. DV: dependent variable. IV: independent variable

Non-clinical changes of the SOC were found over time from T1 to T2 [11], therefore the SOC at T2 was selected as a mediator when the patients were in a relatively stable condition. The mediating effect of SOC was investigated, based on a general mediation model suggested by MacKinnon et al. [49] (Fig. 1). Age (a continuous variable), educational level and cancer stage (dichotomous variables) were controlled for in all analyses as they were significant in the results of our previous study [11]. A series of MLR analyses in three steps, recommended by Baron and Kenny [42], were performed. The first step examined whether the independent variable (T1 scale scores) correlated with the mediator (SOC scale scores). In the second step, it was determined whether the independent variable (T1 scale scores) correlated with the dependent variable (T2 scale scores). The third step examined whether both the mediator (SOC scale scores) and independent variable (T1 scale scores) correlated with the dependent variable (T2 scale scores). As a rule of thumb [42], when the results of the first and second steps of MLR analyses are significant, the degree of SOC is a total mediator if the correlation between the independent (T1 scale scores) and dependent variables (T2 scale scores) is non-significant in the third step. However, the degree of SOC is a partial mediator, if this correlation decreases. The Sobel test was then used on the third step of the MLR analyses to evaluate the significant mediation effect. A value of >1.96 (p <0.05) indicates support for a mediating effect [50]. To further establish the mediation effect, Bootstrapping was used [51]. By Bootstrapping, the mediation effect is estimated based on a large number of Bootstrap samples generated from the original data by random sampling with replacement [51]. In this study Bootstrap was based on 1000 Bootstrap samples with a bias-corrected accelerated method to estimate the 95 % confidence interval. If this estimation does not include zero, it suggests a significant mediation effect at the 0.05 level [52].

For using the MLRs with four independent variables (T1 scale scores, age, educational level and cancer stage) and one mediator (SOC scale score), and selecting a level of R^2^ as small as 0.10 (effect size =0.11), according to Cohen’s Tables [53], a sample size of 154 breast cancer patients is optimal and meets the power of 0.90. Therefore, our sample size (*n* = 162) seems sufficient to test the mediation effect. All assumptions of the MLRs were fulfilled by assessment of the models’ residual [54].

## Results

### Descriptive statistics

Demographic and medical characteristics of the sample are presented in Table 1. The mean age of the breast cancer patients was 46. 1 years (SD = 9.8, range 23–67 years). The mean score of the SOC scale was 63.1 and standard deviation 13.4.

**Table 1:** Demographic and medical characteristics of the sample of women with breast cancer (*n* = 162)

Characteristics	Number	Percent	Characteristics	Number	Percent
Age group (in years)			Cancer stage		
≤49	101	62.3	Mild (stage 0 to II)	121	74.7
>49	61	37.7	Severe (stage III & higher)	41	25.3
Marital status			Chemotherapy		
Single	11	6.8	Yes	128	79.0
Married	129	79.6	No	34	21.0
Divorced/widowed	22	13.6	Radiotherapy		
Education			Yes	122	75.3
High school or less	54	33.3	No	40	24.7
College /university	108	66.7	Hormonal therapy		
Employment status			Yes	111	68.5
Working	51	31.5	No	51	31.5
Not working	111	68.5			
Menopause at baseline					
Yes	60	37.0			
No	102	63.0			
Concomitant disease^a^					
One chronic disease	42	26.0			
More than one	36	22.0			
No disease	84	51.9			
Surgical procedure					
Breast conservation	72	44.4			
Mastectomy	90	55.6			

^a^Long-standing diseases such as diabetes, hypertension, hyperlipidemia, and musculoskeletal problems

### The mediating effect of sense of coherence

The results of bivariate correlations between the mediator (SOC scale scores) and the independent variable (T1 scale scores) as well as between the independent (T1 scale scores) and the dependent variable (T2 scale scores) in the correlation matrix showed that eight pairs out of in total 18 numbers of HRQoL variables were significant to be included in the mediation models. When we controlled the regression models for age, education and disease stage, and the degree of SOC as a mediator was included in the models, the associations between HRQoL dimensions at T1 and the same dimensions at T2 decreased or were no longer significant. The MLRs results from these eight mediation models revealed that the degree of SOC totally mediated variation of the patients’ ratings of global quality of life (p <0.001) as well as CF and SF (p <0.05) scales scores of the EORTC QLQ-C30 from T1 to T2. For the remaining variables, the degree of SOC indicates a partially mediating role: EF (p <0.01), FA (p <0.05), FI (p <0.05), HI (p <0.001) and MCS (p <0.001) scales scores (Table 2). The models with the SOC as a mediator explained 16 % to 45 % of the variances in the T2 scale scores. The Sobel and Bootstrap tests met all criteria for all eight variables, except for the financial scale in the EORTC QLQ-C30 which only met the criterion for the Bootstrap test. In Table 2, the mediating effect of SOC on these eight significant pairs of HRQoL dimensions is presented.

**Table 2:** Testing the mediating effect of sense of coherence (SOC scale scores) in relationship between HRQoL dimensions at baseline (T1 scale scores) and the same dimensions 6 months later (T2 scale scores) in women with breast cancer (*n* = 162) by three steps of multiple linear regression analysis, Sobel test and Bootstrap

	Standardized beta coefficient (b)^b^				Bootstrapped confidence interval (CI)	
Mediation chain: IV^1^ → M^2^ → DV^3^	IV→ M	IV→ DV	IV→ DV M^4^	Sobel test	CI 95 % Lower	CI 95 % Upper
EORTC QLQ-C30 Scales:						
QoL-T1 → SOC-T2 → QoL-T2	.27^a^	.24^b^	.10	3.26^a^	.45	.93
EF-T1 → SOC-T2 → EF-T2	.23^b^	.27^b^	.16^c^	2.72^b^	.11	.27
CF-T1 → SOC-T2 → CF-T2	.23^b^	.16^c^	.09	2.36^c^	.19	.65
SF-T1 → SOC-T2 → SF-T2	.18^c^	.19^c^	.13	2.04^c^	.36	.83
FA-T1 → SOC-T2 → FA-T2	-.17^c^	.25^b^	.19^b^	2.03^c^	-.93	-.42
FI-T1 → SOC-T2 → FI-T2	-.17^c^	.32^a^	.29^a^	1.63	-.88	-.10
Health Index						
HI-T1 → SOC-T2 → HI-T2	.32^a^	.48^a^	.34^a^	3.62^a^	.11	.18
SF-12v2 Scales:						
MCS-T1 → SOC-T2 → MCS-T2	.37^a^	.42^a^	.21^b^	4.36^a^	.37	.60

^a^ *p* < 0.001, ^b^ *p* < 0.01, ^c^ *p* < 0.05

^b^The variables of age, educational level and cancer stage were controlled for in all multiple linear regression analyses

^1^IV, independent variable (HRQoL dimensions at T1)

^2^M, mediator (SOC scale scores at T2)

^3^DV, dependent variable (HRQoL dimensions at T2)

^4^Relationship between IV and DV after controlling for the mediator

## Discussion

This study reveals that the patients’ ratings of HRQoL dimensions before the final diagnosis of breast cancer is related to how they rate the same dimensions 6 months later, and that their degree of SOC mediates these ratings in some dimensions. It is earlier confirmed that cancer patients who report low levels of functions and well-being as well as high levels of symptoms at baseline are significantly at risk for low, respectively high levels of the same dimensions later after treatment [8, 10]. The role of SOC as a mediator of the ratings of HRQoL in cancer patients has scarcely been studied. The degree of SOC has earlier been found to partially mediate between perceived stress and quality of life perception in a sample of women family members of seriously mentally ill adults [55]. The authors suggested that reducing stress and focusing on interventions to strengthen their degree of SOC might assist these women. Hyphantis et al. [56] in a cross-sectional study within a sample of patients diagnosed with systemic lupus erythematosus (SLE) in comparison with a diseased control group reported that the association between psychological distress and physical HRQoL was totally mediated by the degree of SOC in the SLE patients group. The authors concluded that an individual with a higher SOC is more likely to perceive complications as understandable and the treatment as manageable and life as meaningful. It is noteworthy that in our study, the SOC scale score functioned as a total mediator of HRQoL ratings between the pre-diagnosis phase and 6 months later in three dimensions: overall quality of life and cognitive and social functioning. Furthermore, the degree of SOC served as a partial mediator for changes in emotional functioning, fatigue, financial difficulties, well-being, and mental health over time. The partial mediating role of SOC brings to mind that there are probably other factors or mediators that influence the relation between baseline HRQoL dimensions and the same dimensions 6 months later. In a study on quality of life of older women with chronic illness, it was recognized that physical limitation (in areas of symptom bother and functional health) had a significant negative influence on quality of life, and this effect was mediated by two variables, the SOC and illness appraisal [57]. Apart from the level of physical limitation, women with higher SOC and more positive illness appraisal showed higher levels of quality of life. These findings demonstrate that the SOC and positive appraisal may have a protective role to reduce negative effects in the perceived quality of life. Additionally, the relationship between ratings of HRQoL dimensions at two different time points, e.g., from baseline to 6-month later could theoretically explain by a model called “response shift” [58]. This theory discusses that response shift (i.e., time-related changes) might be an important mediator to HRQoL changes and the adaptation process, when patients are confronted with a life-threatening disease [58]. Response shift, suggests that the patients reset their perceptions based on new expectations, standards and values in their life leading to adaption and improved HRQoL [4, 58]. In other words, a response shift is a change in what is important in life as a result of what’s going on in life. Most of the time, the patients develop different values over time as a result of the trajectory of a disease [59]. It is rather natural to hypothesize that the way response-shift appears, depends on the degree of SOC, i.e., how people manage and adjust to life strain, for which we found some support for in our study.

The SOC mediated totally or partially dimensions of psychosocial character on HRQoL ratings more than the dimensions of a physical character, which is consistent with other studies [60, 61]. The question whether the concepts of SOC and mental health are closely interrelated has been raised, but studies indicate that although correlated, they can be considered as independent concepts [17, 22].

The longitudinally mediating effect of the SOC in the context of this study from the pre-diagnosis phase of breast cancer to 6 months later supports the role of SOC as an inner resource, which can positively influence the response to the challenges that are coming with breast cancer over time and reinforce the patient’s recovery in the form of improved quality of life. Here, our findings suggest that a higher degree of SOC may function as a protective mediator for HRQoL dimensions in the process of psychological adaptation to the cancer trajectory.

Testing the mediating role of the SOC in a longitudinal design enabled us to find a new perspective to the influence of the baseline HRQoL variables on the same variables 6 months later. The SF-12v2 and HI as generic HRQoL measurements were used in this study to confirm the results of the disease-specific questionnaire EORTC QLQ-C30, as they might measure different perspectives. Our findings showed that the results were consistent in their parallel respective scales of overall health status, physical, and mental/emotional dimensions.

In Summary, the findings of our study add to the knowledge about the longitudinally role of the SOC in the context of coping with breast cancer before final diagnosis and 6 months later during a time of complex treatment decisions or treatment end. The present findings lend some support to Antonovsky’s theory about the SOC and coping. The SOC can be viewed as a “resistance resource” that helps individuals experience stress as less threatening, to cope with it more efficiently, and to experience less stress-related illness [16]. The structures embedded in the concept of SOC, comprehensibility, manageability, and meaningfulness, showed a buffering effect on perceived HRQoL impairments. Therefore, considering the coherence-oriented structure of the SOC is important. Integrating knowledge of patients’ HRQoL and their perceptions of a how comprehensible, manageable and meaningful their situation is, may provide a foundation to detect the women who are at greater risk for psychological maladaptation to the disease trajectory and HRQoL impairment.

## Conclusions

This study shows that the ratings of some of the dimensions of HRQoL by breast cancer patients are longitudinally mediated by the way how they view their life as comprehensible, manageable and meaningful, and thereby cope with life strain, in this study measured by the Sense of Coherence Scale. The mediating pathway of the sense of coherence in the context of this study appears to be the key to understanding how a higher sense of coherence as an inner resource may serve as a protective psychological factor in the adaptation to breast cancer and consequently HRQoL impairments over time. This indicates the importance to focus on the identification of potential problem areas of HRQoL in relation to the patient´s degree of sense of coherence to incorporate them in intervention plans from the first visit at pre-diagnosis period onwards.

## Abbreviations

HRQoL: Health-related quality of life
SOC: Sense of coherence
GRR: Generalized resistance resource
GRD: Generalized resistance deficit
EORTC: European Organization for research and treatment of cancer
SF-12v2: SF-12 Health survey version 2
HI: Health index
PF: Physical functioning
RF: Role functioning
EF: Emotional functioning
CF: Cognitive functioning
SF: Social functioning
FA: Fatigue
PA: Pain
NV: Nausea/vomiting
AP: Appetite loss
SL: Insomnia
DY: Dyspnea
CO: Constipation
DI: Diarrhea
FI: Financial difficulties
PCS: Physical component summary
MCS: Mental component summary
MLR: Multiple linear regression
